# Prolonged Persistence of SARS-CoV-2 RNA in Body Fluids

**DOI:** 10.3201/eid2608.201097

**Published:** 2020-08

**Authors:** Jiufeng Sun, Jianpeng Xiao, Ruilin Sun, Xi Tang, Chumin Liang, Huifang Lin, Lilian Zeng, Jianxiong Hu, Runyu Yuan, Pingping Zhou, Jinju Peng, Qianlin Xiong, Fengfu Cui, Zhe Liu, Jing Lu, Junzhang Tian, Wenjun Ma, Changwen Ke

**Affiliations:** Guangdong Provincial Center for Disease Control and Prevention, Guangzhou, China (J. Sun, J. Xiao, C. Liang, H. Lin, L. Zeng, J. Hu, R. Yuan, P. Zhou, J. Peng, Q. Xiong, F. Cui, Z. Liu, J. Lu, W. Ma, C. Ke);; Guangdong Second Provincial General Hospital, Guangzhou (R. Sun, J. Tian);; First People’s Hospital of Foshan, Foshan, China (X. Tang)

**Keywords:** SARS-CoV-2, RNA, COVID-19, persistence, coronavirus, Guangdong, China, respiratory infections, viruses, 2019 novel coronavirus disease, severe acute respiratory syndrome coronavirus 2, zoonoses, coronavirus disease

## Abstract

We prospectively assessed 49 coronavirus disease cases in Guangdong, China, to estimate the frequency and duration of detectable severe acute respiratory syndrome coronavirus 2 RNA in human body fluids. The prolonged persistence of virus RNA in various body fluids may guide the clinical diagnosis and prevention of onward virus transmission.

In December 2019, coronavirus disease (COVID-19) caused by a novel coronavirus, severe acute respiratory syndrome coronavirus 2 (SARS-CoV-2), emerged in Wuhan, China (*1*,*2*). As of April 1, 2020, the virus had expanded to 195 countries, and >820,000 confirmed cases with >40,000 deaths had been recorded (*3**–**6*). 

Clinically, the confirmation of SARS-CoV-2 infection relies on detection of virus RNA in various body fluids. The World Health Organization recommends taking upper and lower respiratory samples simultaneously during the acute phase of infection to detect virus RNA. Recent studies reported a persistent shedding of SARS-CoV-2 in upper respiratory and intestinal samples (*7*,*8*). However, the frequency with which SARS-CoV-2 RNA can be detected in body fluids and the period during which it remains detectable are not well understood. A detailed understanding of the dynamics of the early stages of SARS-CoV-2 infection is needed to inform diagnostic testing and prevention interventions, because existing evidence is based only on observations from case reports. We recruited hospitalized patients with COVID-19 from 2 designated provincial emergency hospitals for emerging infectious diseases in Guangdong, China, and tested specimens by real-time reverse transcription PCR (rRT-PCR) to estimate the duration of the detection of SARS-CoV-2 RNA in various body fluids, using an accelerated failure time (AFT)–based modeling study.

## The Study

We recruited 43 patients with mild cases of COVID-19 (22 male, 21 female; median age 43, range 1–70 years) and 6 patients with severe cases (6 male; median age 67, range 46–76 years) for this study. We obtained throat swab, nasopharyngeal swab, sputum, and feces specimens every 3 days for 4 weeks. We tested all specimens by rRT-PCR (Appendix). We used parametric Weibull regression models (AFT) to estimate the time until the loss of SARS-CoV-2 RNA detection in each body fluid and reported findings in medians and 95th percentiles using R software version 3.6.1 with flexsurv, survival, and survminer packages (*9*). We used Lnorm and gamma models as comparisons to evaluate the sensitivity and stability of Weibull regression models. We defined the time until loss of SARS-CoV-2 RNA detection in each fluid as the number of days between the day after illness onset and the day of the first negative rRT-PCR result. For the cases that involved intermittent shedding of SARS-CoV-2, we used the date of the first negative result after the final recorded positive rRT-PCR results. Of the 49 case-patients, 15 were discharged from the hospital after <4 weeks of observation time. 

**Figure 1:**
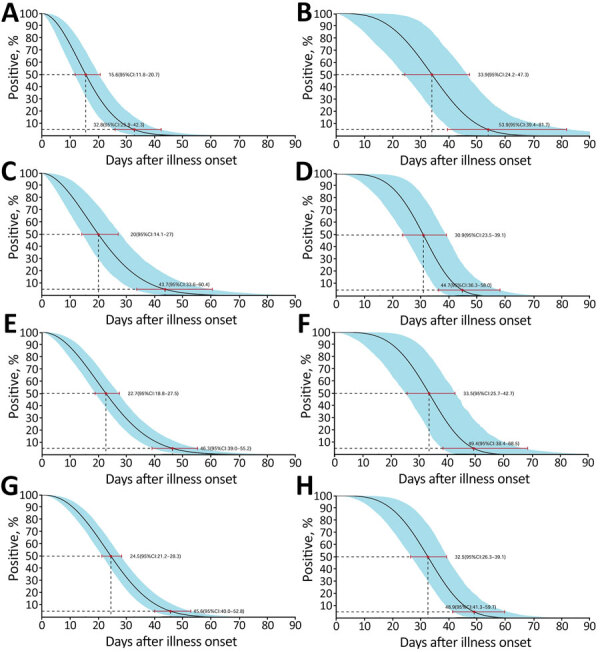
Time until clearance of severe acute respiratory syndrome coronavirus 2 RNA in throat swab, sputum, nasopharyngeal, and feces samples among hospitalized patients with coronavirus disease, as estimated with the use of Weibull regression, Guangdong, China. A, B) Throat swab specimens from patients with mild (A) and severe (B) cases; C, D) sputum samples from patients with mild (C) and severe (D) cases; nasopharyngeal swab samples from patients with mild (E) and severe (F) cases; G, H) feces samples from patients with mild (G) and severe (H) cases. A total of 43 patients with mild and 6 with severe cases were tested. The medians and 95th percentiles of the time until the loss of detection are indicated; error bars and shading indicate 95% CIs.

**Figure 2:**
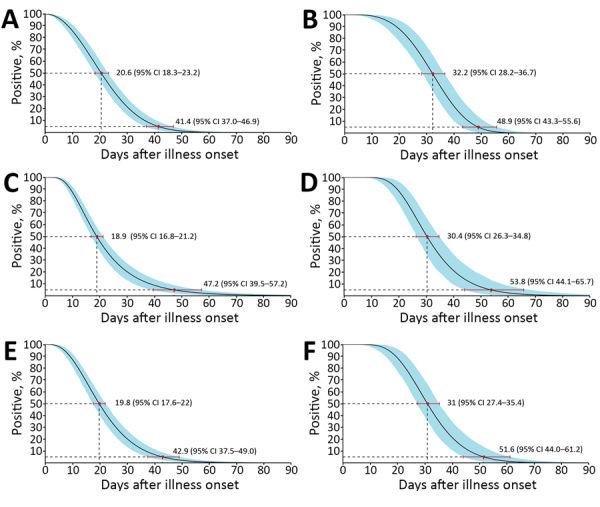
Time until clearance of severe acute respiratory syndrome coronavirus 2 RNA in any clinical specimens of throat swab, sputum, nasopharyngeal swab, or feces samples among hospitalized patients with coronavirus disease, as estimated with the use of Weibull, Lnorm, and gamma regression, Guangdong, China. A, B) Weibull regression for mild (A) and severe (B) cases; C, D) Lnorm regression for mild (C) and severe cases (D); E, F) gamma regression for mild (E) and severe (F) cases. A total of 43 patients with mild and 6 with severe cases were tested. The medians and 95th percentiles of the time until the loss of detection are indicated; error bars and shading indicate 95% CIs.

We obtained a total of 490 specimens (32.75% of the designated number of samples, 1,006 missing samples), including 88 throat swab samples (23.53%, 198 missing samples), 62 sputum samples (16.58%, 312 missing samples), 175 nasopharyngeal swab samples (46.79%, 199 missing samples), and 165 fecal samples (44.12%, 209 missing samples). Of these, 171 specimens tested positive for SARS-CoV-2 RNA by rRT-PCR, including 16 throat swab samples, 38 sputum samples, 89 nasopharyngeal swab samples, and 28 feces samples (Appendix Figure 1). We used Weibull models to estimate the median and the 95th percentile for the time until the loss of SARS-CoV-2 RNA detection in swab, sputum, and fecal samples (Table; Figures 1, 2). The sensitivity and stability evaluation of the Weibull, Lnorm, and gamma models showed no differences among them (p<0.05) (Appendix Table, Figures 2, 3).

**Table:** Prolonged persistence of SARS-CoV-2 RNA in body fluids from hospitalized patients with coronavirus disease, Guangdong, China*

Specimens	Mild cases, n = 43			Severe cases, n = 6	
	Median (95% CI)	95th percentile (95% CI)		Median (95% CI)	95th percentile (95% CI)
Throat swab	15.6 (11.8–20.7)	32.8 (25.9–42.3)		33.9 (24.2–47.3)	53.9 (39.4–81.7)
Sputum	20.0 (14.1–27.0)	43.7 (33.6–60.4)		30.9 (23.5–39.1)	44.7 (36.3–58.0)
Nasopharyngeal swab	22.7 (18.8–27.5)	46.3 (39.0–55.2)		33.5 (25.7–42.7)	49.4 (38.4–68.5)
Feces	24.5 (21.2–28.3)	45.6 (40.0–52.8)		32.5 (26.3–39.1)	48.9 (41.3–59.7)

*The time until the loss of SARS-CoV-2 RNA detection in each body fluid was estimated by using parametric Weibull regression models. Data are presented as medians and 95th percentiles in days after illness onset. SARS-CoV-2, severe acute respiratory syndrome coronavirus 2.

## Conclusions

In this study, we estimated the time for COVID-19 case-patients to clear SARS-CoV-2 RNA in the acute phase of infection through an AFT-based modeling study. We found persistent shedding of virus RNA in nasopharyngeal swab and feces samples. The estimated time until loss of virus RNA detection ranged from 45.6 days for nasopharyngeal swab samples to 46.3 days for feces samples in mild cases and from 48.9 days for nasopharyngeal swab samples to 49.4 days for feces samples in severe cases, which was longer than those of SARS-CoV and MERS-CoV (*10*,*11*). Lan et al. reported positive rRT-PCR results in throat swab sampless from patients who recovered from mild COVID-19 for 50 days at maximum (*8*). Wu et al. found prolonged presence of SARS-CoV-2 viral RNA in fecal samples (*7*). However, we found that the median time for throat samples from mild cases was 15.6 days (95% CI 11.8–20.7 days) and the 95th percentile was 32.8 days (95% CI 25.9–42.3 days). Therefore, detection of virus RNA for mild cases in throat swab samples at the 50th day after illness onset should be a low-probability event, beyond the 95th percentile limit. Similarly, the detection of virus RNA in fecal samples from mild cases was also close to the 95th percentile limit we estimated (45.6 days, 95% CI 40.0−52.8 days). 

We found differences in median time until loss of virus RNA detection among respiratory specimen types in mild cases but not in severe cases (Table). We do not believe this finding was linked to the severity of COVID-19, but we had a limited sample size of severe cases in this study. The additional test using Lnorm or gamma models addressed similar phenomena. Nevertheless, the estimated time until the loss of RNA detection in various body fluids in this study was reasonable and was consistent with previous findings in case reports.

Challenges have been raised recently in the molecular diagnosis of COVID-19. Upper respiratory samples show lower positive rates and instable states of confirmation of SARS-CoV-2, whereas lower respiratory samples, such as bronchoalveolar lavage fluid, are suitable specimens for detection of virus RNA (*12*). The probable explanation of discrepant results with our estimates was the irregular operation of sampling in upper respiratory samples in clinics, rather than short-term shedding of virus RNA. In addition, the median duration in archived publications in China was 12.0 days (mean 12.8 days) (*13*), which was shorter than, but close to, our estimate in throat swab samples. This finding was in line with throat swab samples being suggested as a clinical sample for diagnosis of COVID-19 at the early stages of the outbreak in China (National Health Commission of the People’s Republic of China, unpub. data, 2020 Jan 15). 

Our study has limitations. First, we did not test serum specimens to address RNAemia or serologic trends. The reasons are the findings of extreme low positive rates of RNAemia in our initial study (1 of 49 cases), which does not yield any estimated conclusion. The serologic test was not conducted because reliable IgM/IgG kits were unavailable. Second, virus isolation and tests of specimens’ infectivity were not conducted. We focused on estimating the duration of the detection of SARS-CoV-2 RNA in various body fluids among COVID-19 cases but did not imply the existence of infectious virus particles. Third, the number of missing specimens was higher than the initial study designed, attributed mainly to low proportions of purulent sputum production in viral pneumonia cases, as well as low compliance of patients. Fourth, this study may pose selection bias because modest-sized groups of cases were included. Finally, the prerequisite we assumed was that all COVID-19 cases had SARS-CoV-2 RNA in all sampling specimens at symptom onset, which means that the median and 95th percentile we estimated were shorter than expected because of the uncertainty of incubation time. The time estimated in this study through hospitalized COVID-19 cases may not be generalizable to all infections with SARS-CoV-2, such as asymptomatic cases.

In conclusion, our results show prolonged persistence of SARS-CoV-2 RNA in hospitalized patients with COVID-19. Health professionals should consider these findings in diagnostic recommendations and prevention measures for COVID-19.

Appendix Additional information about testing for persistence of SARS-SoV-2 RNA in body fluids.

## References

[1] Wu F, Zhao S, Yu B, Chen YM, Wang W, Song ZG, et al. A new coronavirus associated with human respiratory disease in China. Nature. 2020;579:265–9. 10.1038/s41586-020-2008-3 32015508 PMC7094943

[2] Zhou P, Yang XL, Wang XG, Hu B, Zhang L, Zhang W, et al. A pneumonia outbreak associated with a new coronavirus of probable bat origin. Nature. 2020;579:270–3. 10.1038/s41586-020-2012-7 32015507 PMC7095418

[3] Li Q, Guan X, Wu P, Wang X, Zhou L, Tong Y, et al. Early transmission dynamics in Wuhan, China, of novel coronavirus-infected pneumonia. N Engl J Med. 2020;382:1199–207. 10.1056/NEJMoa2001316 31995857 PMC7121484

[4] Holshue ML, DeBolt C, Lindquist S, Lofy KH, Wiesman J, Bruce H, et al.; Washington State 2019-nCoV Case Investigation Team. First case of 2019 novel coronavirus in the United States. N Engl J Med. 2020;382:929–36. 10.1056/NEJMoa2001191 32004427 PMC7092802

[5] Rothe C, Schunk M, Sothmann P, Bretzel G, Froeschl G, Wallrauch C, et al. Transmission of 2019-nCoV infection from an asymptomatic contact in Germany. N Engl J Med. 2020;382:970–1. 10.1056/NEJMc2001468 32003551 PMC7120970

[6] Wu JT, Leung K, Leung GM. Nowcasting and forecasting the potential domestic and international spread of the 2019-nCoV outbreak originating in Wuhan, China: a modelling study. Lancet. 2020;395:689–97. 10.1016/S0140-6736(20)30260-9 32014114 PMC7159271

[7] Wu Y, Guo C, Tang L, Hong Z, Zhou J, Dong X, et al. Prolonged presence of SARS-CoV-2 viral RNA in faecal samples. Lancet Gastroenterol Hepatol. 2020;5:434–5. 10.1016/S2468-1253(20)30083-2 32199469 PMC7158584

[8] Lan L, Xu D, Ye G, Xia C, Wang S, Li Y, et al. Positive RT-PCR test results in patients recovered from COVID-19. JAMA. 2020;2020:27. 10.1001/jama.2020.2783 32105304 PMC7047852

[9] Paz-Bailey G, Rosenberg ES, Doyle K, Munoz-Jordan J, Santiago GA, Klein L, et al. Persistence of Zika virus in body fluids—final report. N Engl J Med. 2017;379:1234–43. 10.1056/NEJMoa1613108 28195756 PMC5831142

[10] Oh MD, Park WB, Choe PG, Choi SJ, Kim JI, Chae J, et al. Viral load kinetics of MERS coronavirus infection. N Engl J Med. 2016;375:1303–5. 10.1056/NEJMc1511695 27682053

[11] Corman VM, Albarrak AM, Omrani AS, Albarrak MM, Farah ME, Almasri M, et al. Viral shedding and antibody response in 37 patients with Middle East respiratory syndrome coronavirus infection. Clin Infect Dis. 2016;62:477–83. 26565003 10.1093/cid/civ951 PMC7108065

[12] Zhang W, Du RH, Li B, Zheng XS, Yang XL, Hu B, et al. Molecular and serological investigation of 2019-nCoV infected patients: implication of multiple shedding routes. Emerg Microbes Infect. 2020;9:386–9. 10.1080/22221751.2020.1729071 32065057 PMC7048229

[13] Guan WJ, Ni ZY, Hu Y, Liang WH, Ou CQ, He JX, et al.; China Medical Treatment Expert Group for Covid-19. Clinical characteristics of coronavirus disease 2019 in China. N Engl J Med. 2020;382:1708–20. 10.1056/NEJMoa2002032 32109013 PMC7092819

