# Supplementary material for: Prolonged Persistence of SARS-CoV-2 RNA in Body Fluids
Source: Emerg Infect Dis. 2020 Aug;26(8):1834–8. doi: 10.3201/eid2608.201097 (PMC7392422; doi:10.3201/eid2608.201097)
Supplement: Appendix — Additional information about testing for persistence of SARS-SoV-2 RNA in body fluids. [file 20-1097-Techapp-s1.pdf]

Article DOI: https://doi.org/10.3201/eid2608.201097

Prolonged Persistence of SARS-CoV-2 RNA in Body Fluids

Appendix

Materials and Methods

Ethics Approval

The novel coronavirus virus disease (COVID-19) was considered part of an ongoing emerging public health outbreak event in China. Data collection and analysis of cases and close contacts were considered exempt from institutional review board approval for center of disease control and prevention in China.

Epidemiology Information Collection

Beginning on January 23, 2020, we prospectively recruited COVID-19 case-patients from the Guangdong Second Provincial General Hospital and the First Hospital of Foshan in Guangdong, China, 2 designated Guangdong provincial emergency hospitals for emerging infectious diseases. All the recruited COVID-19 case-patients were confirmed for SARS-CoV-2 infection through rRT-PCR assay. COVID-19 case-patients transferred to other hospitals after hospitalization in these 2 hospitals were excluded from this study. Cases beyond the observation time because of different dates of illness onset, when they were enrolled or discharged before 4 weeks' observation, were also included for modeling estimation. Demographic information and dates of illness onset were collected from all recruited COVID-19 cases according to the standard epidemiology investigation workflow in those 2 hospitals.

Case Definitions and Criteria for Discharge from Hospital

A clinical suspect case was in a patient who had a travel history to Wuhan or direct contact with patients from Wuhan who had fever or respiratory symptoms, within 14 days before illness onset. A laboratory-confirmed case was defined as a case in a patient with respiratory specimens who tested positive for the SARS-CoV-2 by $\geq$1 of the following 3 methods: isolation of virus or $\geq$2 positive results by rRT-PCR assay for SARS-CoV-2 or a genetic sequence that

matches SARS-CoV-2.The patients were categorized into severe and mild cases depending on whether they were admitted to an intensive care unit or received oxygen supplementation and ventilation treatment during the hospitalization.

The criteria for hospital discharge in China were issued on guidelines for COVID-19 clinical diagnosis and treatment (version I–VI). Patients with COVID-19 who showed absence of clinical symptoms, whose body temperature was normal for >3 days, and whose lung computed tomography improved notably, without any acute manifestations such as exudation, and had with 2 serial negative RT-PCR test results (24 h interval) could be discharged from the hospital.

Specimen Collection and Storage

Specimen collection from COVID-19 case-patients was conducted in consultation with a healthcare provider. For nasopharyngeal specimens, a swab was inserted into the nostril parallel to the palate to a depth equal to the distance from the nostrils to the outer opening of the ear. The swab was left in place for several seconds to absorb secretions and then was slowly removed with rotation. For throat samples, the posterior pharynx was swabbed, avoiding the tongue. For sputum samples, the patient was asked to rinse the mouth with water and then expectorate a deep cough sputum directly into a sterile, leakproof, screw-cap collection cup. Feces samples were also collected in screw-cap collection cups. Specimens were immediately stored at 2–8°C and transported under the same conditions to the Guangdong Provincial Center for Disease Control and Prevention for viral RNA extraction and molecular testing. Residual specimens were stored at −70°C or below.

Nucleic Acid Extraction and rRT-PCR

The swab samples were vortexed in 2.5 mL of commercial viral transport media and 200 μL was recovered for RNA extraction. For fecal samples, ≈10 g of feces were added to a tube with 10 mL of phosphate-buffered saline (PBS) and glass beads. After vortexing at 2000 rpm for 10 min, the same volume (200 μL) of supernatant was recovered for RNA extraction. For sputum samples, the sputum was added to a tube with 5 mL of PBS and glass beads. After vortexing at 2000 rpm for 10 min, we recovered the same volume of supernatant for RNA extraction.

We extracted total RNA using a prefilled viral total NA kit-Flex (Fisher Scientific, Labserv, Cat.No. KFRPF-805296; https://www.fishersci.com) following manufacturer's instructions. All samples were lysed, and the nucleic acids bound to the surface of magnetic

beads. The beads were washed with buffers to remove residual proteins and contaminants and the purified nucleic acids were eluted with 50 μL of nuclease free water for subsequent testing.

A commercial rRT-PCR assay kit targeting the ORF1ab and N genes was used to detect SARS-CoV-2 RNA (DaAn Gene, Guangzhou, China, cat. no. DA0931; http://en.daangene.com). Amplification was performed on an Applied Biosystems 7500 Real-Time PCR System (ThermoFisher Scientific, https://www.thermofisher.com) as follows: 50°C for 15 min, 95°C for 15 min, followed by 45 cycles of 94°C for 15s and 55°C for 45s. Specimens were considered positive for SARS-CoV-2 RNA if both ORF1ab and N gene target amplification curves were generated within 40 cycles.

Modeling Estimates

We fit separate parametric accelerated failure time (AFT) survival models (Weibull, Lnorm, and gamma) to the outcomes of the time to loss of RNA detection in throat swabs, nasopharyngeal swabs, sputum samples, and feces specimens. The outcome of time to loss of RNA detection in each fluid was defined as the days until the first negative RT-PCR result. For those shedding intermittently, we used the first negative result after the final recorded RT-PCR-positive test result. Survival time estimates at given quantiles of cumulative survival (50th and 95th percentiles) by using standard maximum-likelihood estimation approaches.

Appendix Table. Prolonged persistence of SARS-CoV-2 in body fluids.

		Mild cases (n = 43)		Severe cases (n = 6)	
Model	Specimens	Median (95% CI)	95th percentile (95% CI)	Median (95% CI)	95th percentile (95% CI)
Gamma	Throat swab	15.3 (12.0–19.1)	31.1 (24.0–40.9)	31.9 (20.7–46.2)	61.3 (39.6–102.0)
	Sputum	19.6 (14.9–25.7)	40.8 (29.3–56.4)	29.9 (24.1–37.2)	45.4 (34.8–61.9)
	Nasopharyngeal swab	21.8 (18.4–25.5)	47.4 (39.3–57.3)	32.5 (25.5–40.9)	51.6 (37.3–76.0)
	Feces	23.3 (20.2–26.9)	49.0 (41.3–59.3)	31.3 (25.7–37.8)	51.0 (40.8–66.2)
Lnorm	Throat swab	14.7 (11.8–18.3)	31.6 (23.3–45.4)	30.9 (19.8–46.9)	67.8 (37.6–138.0)
	Sputum	18.8 (14.7–23.7)	40.0 (28.5–61.2)	29.6 (23.8–36.3)	46.0 (35.3–66.5)
	Nasopharyngeal swab	20.8 (17.7–25.1)	51.3 (39.8–67.3)	32.0 (24.5–41.4)	53.5 (36.8–91.6)
	Feces	22.3 (19.2–26.0)	55.2 (43.8–70.6)	30.7 (25.2–37.7)	52.7 (39.9–74.0)

*The time until the loss of SARS-CoV-2 RNA detection in each body fluid was estimated by using parametric gamma and Lnorm regression models. The data are presented as medians and 95th percentiles since days after illness onset.

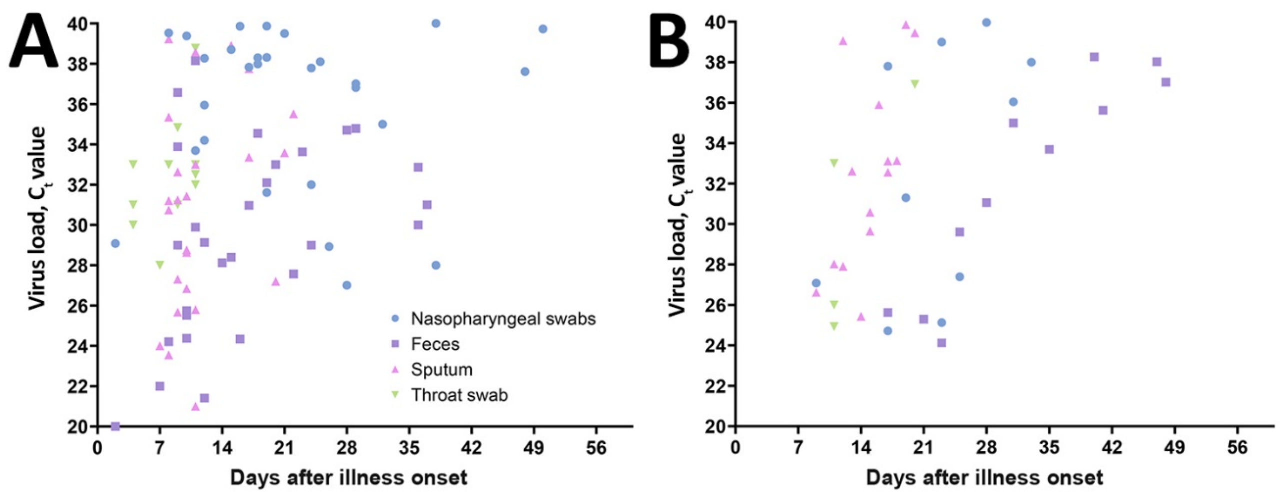

Appendix Figure 1. Specimens were considered positive if target amplification was detected within 40 amplification cycles. Viral loads were expressed as the cycle threshold (C_t) value of rRT-PCR. Results are presented for A) mild and B) severe COVID-19 cases in throat swabs, sputum, nasopharyngeal swabs, and feces samples.

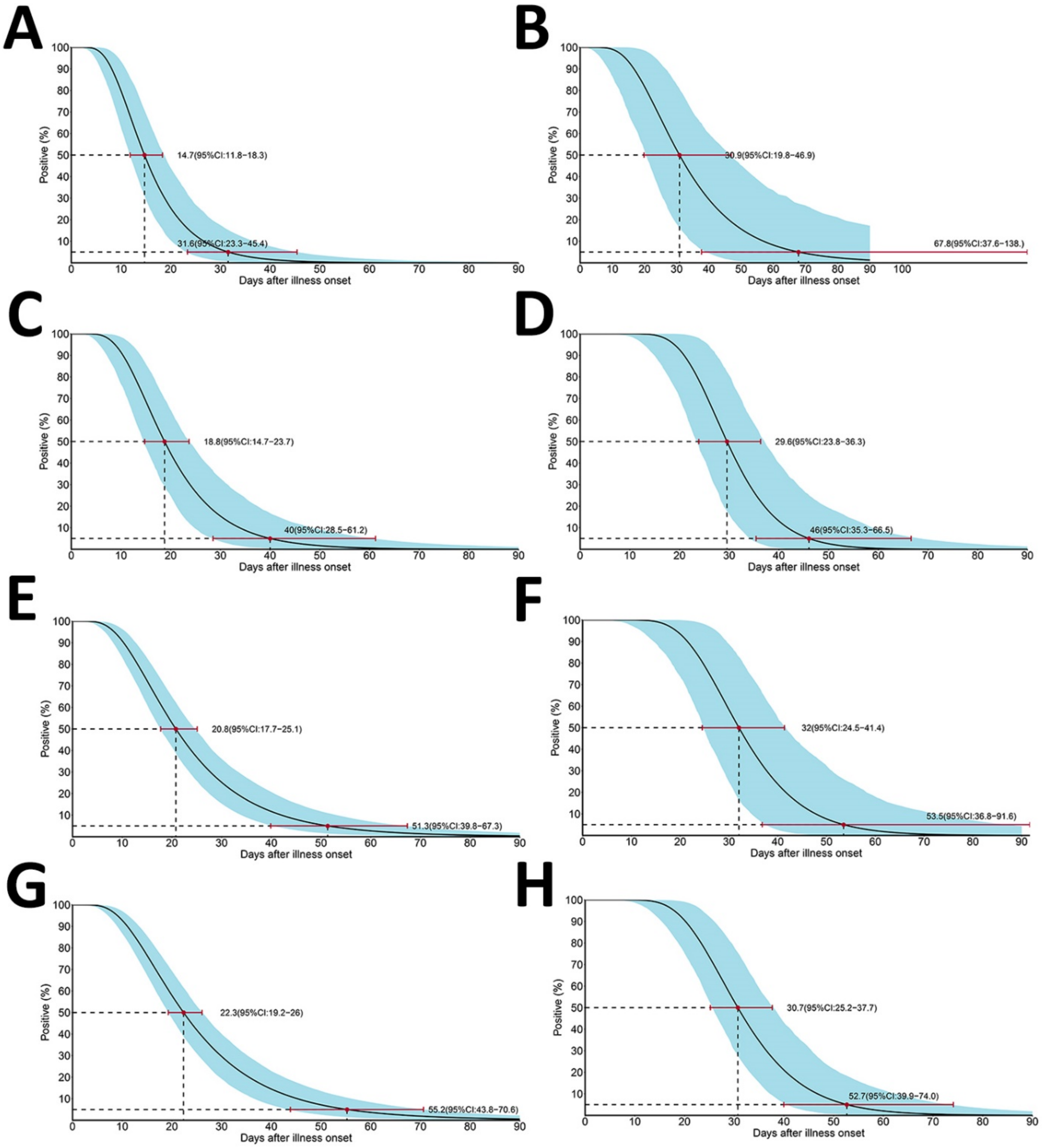

Appendix Figure 2. Time until the clearance of SARS-CoV-2 RNA in throat swabs, sputum, nasopharyngeal swabs, and feces using an Lnorm regression model. Shown are the time until the loss of SARS-CoV-2 RNA detection after the onset of symptoms in throat swabs (A, mild cases; B, severe cases), sputum (C, mild cases; D, severe cases), nasopharyngeal swabs (E, mild cases; F, severe cases), and feces (G, mild cases; H, severe cases) from 43 mild and 6 severe COVID-19 cases in hospitalized patients. The medians and 95th percentiles of the time until the loss of detection is shown in each panel with 95% confidence intervals (blue shading).

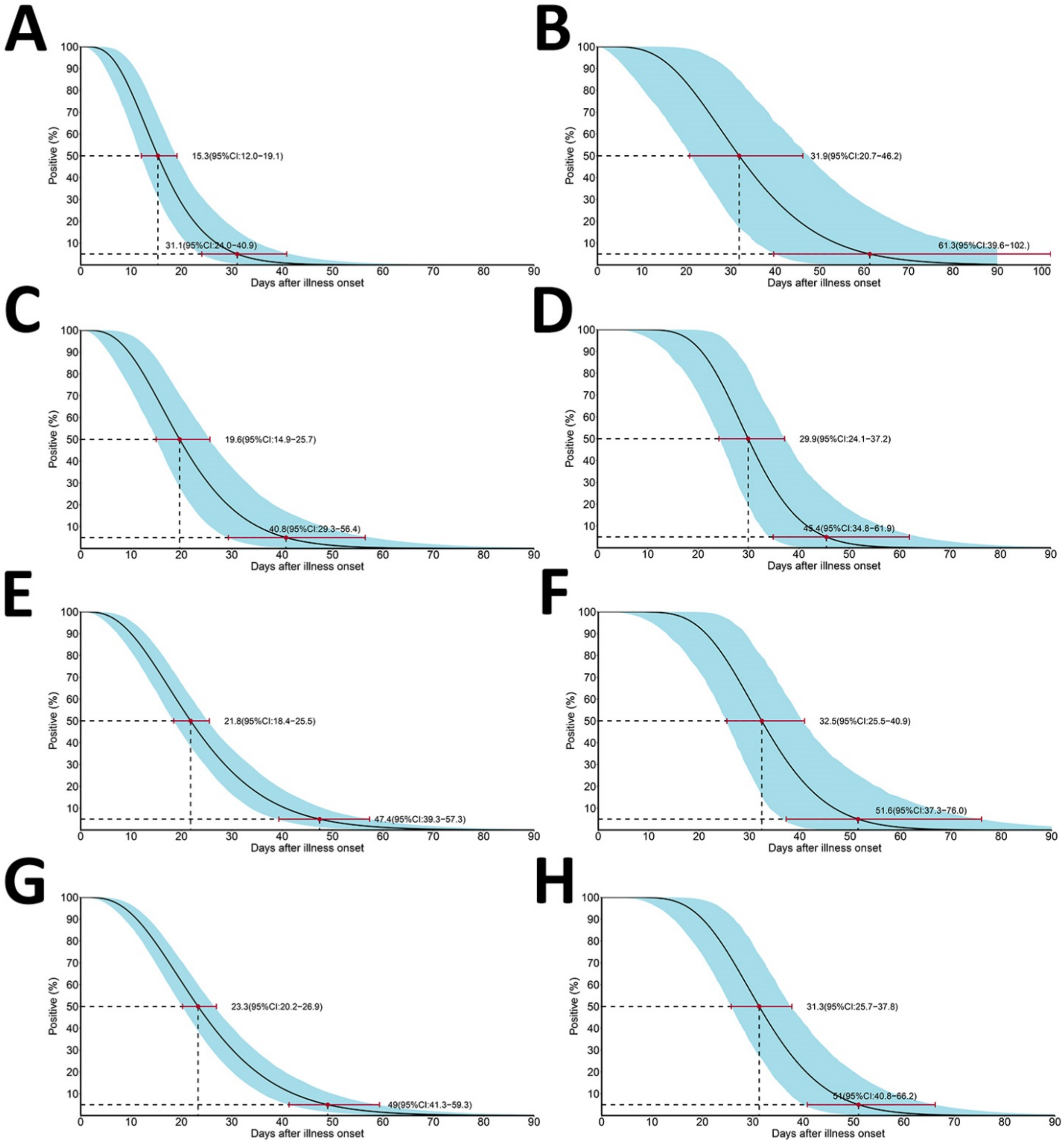

Appendix Figure 3. Time until the clearance of SARS-CoV-2 RNA in throat swabs, sputum, nasopharyngeal swabs, and feces by using a gamma regression model. Shown are the time until the loss of SARS-CoV-2 RNA detection after the onset of symptoms in throat swabs (A, mild cases; B, severe cases), sputum (C, mild cases; D, severe cases), nasopharyngeal swabs (E, mild cases; F, severe cases), and feces (G, mild cases; H, severe cases) from 43 mild and 6 severe COVID-19 cases in hospitalized patients. The medians and 95th percentiles of the time until the loss of detection is shown in each panel with 95% confidence intervals (blue shading).
